# *Lactobacillus plantarum* FRT4 alleviated obesity by modulating gut microbiota and liver metabolome in high-fat diet-induced obese mice

**DOI:** 10.29219/fnr.v66.7974

**Published:** 2022-05-09

**Authors:** Hongying Cai, Zhiguo Wen, Lulu Zhao, Dali Yu, Kun Meng, Peilong Yang

**Affiliations:** 1 Institute of Feed Research, Chinese Academy of Agricultural Sciences, Beijing, China; 2 National Engineering Research Center of Biological Feed, Beijing, China; 3 School of Life Sciences, Qilu Normal University, Jinan, P. R. China

**Keywords:** Lactobacillus plantarum FRT4, obesity, liver metabolomics, glycerophospholipid metabolism, gut microbiota

## Abstract

**Background:**

Obesity has become a global epidemic recognized by the World Health Organization. Probiotics supplementation has been shown to contribute to improve lipid metabolism. However, mechanisms of action of probiotics against obesity are still not clear. *Lactobacillus plantarum* FRT4, a probiotic previously isolated from a kind of local yogurt, had good acid and bile salt tolerance and lowered cholesterol *in vitro*.

**Objective:**

This study aimed to evaluate the effect of *L. plantarum* FRT4 on serum and liver lipid profile, liver metabolomics, and gut microbiota in mice fed with a high-fat diet (HFD).

**Design:**

Mice were fed with either normal diet or HFD for 16 weeks and administered 0.2 mL of 1 × 10^9^ or 1 × 10^10^ CFU/mL dosage of *L. plantarum* FRT4 during the last 8 weeks of the diet. Cecal contents were analyzed by 16S rRNA sequencing. Hepatic gene expression and metabolites were detected by real-time quantitative polymerase chain reaction (PCR) and metabolomics, respectively.

**Results:**

*L. plantarum* FRT4 intervention significantly reduced the HFD-induced body weight gain, liver weight, fat weight, serum cholesterol, triglyceride, and alanine aminotransferase (ALT) levels in the liver (*P <* 0.05). Liver metabolomics demonstrated that the HFD increased choline, glycerophosphocholine, and phosphorylcholine involved in the glycerophospholipid metabolism pathway. All these changes were reversed by FRT4 treatment, bringing the levels close to those in the control group. Further mechanisms showed that FRT4 favorably regulated gut barrier function and pro-inflammatory biomediators. Furthermore, FRT4 intervention altered the gut microbiota profiles and increased microbial diversity. The relative abundances of *Bacteroides*, *Parabateroides*, *Anaerotruncus*, *Alistipes*, *Intestinimonas*, *Butyicicoccus*, and *Butyricimonas* were significantly upregulated. Finally, Spearman’s correlation analysis revealed that several specific genera were strongly correlated with glycerophospholipid metabolites (*P <* 0.05).

**Conclusions:**

These findings suggested that *L. plantarum* FRT4 had beneficial effects against obesity in HFD-induced obese mice and can be used as a potential functional food for the prevention of obesity.

## Popular scientific summary

- *Lactobacillus plantarum* FRT4, isolated from a kind of local yogurt in Xinjiang province without antimicrobial-resistant genes, decreased body weight gain, liver weight, fat weight, serum cholesterol, triglyceride, and ALT levels in liver.
- FRT4 treatment reshaped the gut microbiota, and upregulated the genera of *Bacteroides, Parabateroides, Anaerotruncus, Alistipes, Intestinimonas, Butyicicoccus*, and *Butyricimonas*.
- FRT4 treatment significantly restored the levels of choline, glycerophosphocholine, and phosphorylcholine to levels similar to those in the normal group.

Obesity is a common chronic metabolic disease in many developed and developing countries that raise the risk for type 2 diabetes, cardiovascular disease, and hepatic steatosis (1). It is widely believed that unhealthy diets, sedentary lifestyles, and sleep deprivation are linked to the rise in obesity. At present, the methods to treat obesity mainly include diet regulation, exercise, surgical treatment, and drug regulation. Among these, surgery and drug regulation have been shown to be related to severe side effects (2). Therefore, searching for an effective method of obesity management has attracted extensive research interest.

Accumulating studies have shown that the gut microbiota is an important environmental factor contributing to obesity by affecting host energy harvest and storage (3). Evidence has revealed that gut microbiota is closely related to host physiology, such as obesity and related metabolic disorders (4). Decreased diversity of gut microbiota and increased endotoxin-producing bacteria have been shown to contribute to the development of nonalcoholic fatty liver disease (NAFLD) (5). Accumulating evidence indicates that the gut microbiota may be a potential therapeutic target for treating metabolic disease (6). Treatment of probiotics can ameliorate the dysbiosis-linked host metabolic changes observed in obesity and related disorders (7). *Lactobacillus plantarum* is widely used as probiotics due to its natural habitation in the human intestinal tract (8) and has a variety of functional properties, such as cholesterol-lowering effect, antioxidant activity, and immune regulation (9, 10). Increasing studies of *L. plantarum* on improving markers of metabolic dysfunction by regulating gut microbiota in high-fat diet (HFD)-induced obese mice have been reported (11, 12). The liver has a supply of arterial and venous blood, with most of the hepatic blood flow coming from the intestine *via* the portal vein. The gut that is compromised by dysbiosis is a portal for increased exposure of the liver to bacteria, bacterial products, and injurious components of foods that contribute to the pathogenesis of obesity-related disorders (5). Numerous studies have shown that *L. plantarum* can improve lipid metabolism and alleviate obesity induced by HFD in animal models. *L. plantarum* FRT10 alleviated obesity in mice by activating the PPARα/CPT1α pathway (13). *L. plantarum* HT121 and *L. plantarum* FZU3013 improved lipid metabolism by increased expression of bile secretion-related genes cholesterol 7α-hydroxylase (*Cyp7*α*1*) and altering bile acid metabolism (14, 15). *L. plantarum* CQPC01 could alleviate inflammation through decreased level of pro-inflammatory factors and increased level of interleukin-4 (IL-4) and interleukin-10 (IL-10) in serum (16). *L. plantarum* Y44 upregulated the expression of occludin and claudin-1 in colon and downregulated the expression of serum interleukin-8 (IL-8) and tumor necrosis factor-α (TNF-α) (17). Although increasing strains of *L. plantarum* have been reported to exhibit protective effects on reducing obesity, the metabolic mechanism involved in lipid-lowering capacity of *Lactobacillus* has not been well elucidated, and few studies have comprehensively investigated the impact of *L. plantarum* on gut microbiota associated with liver metabolomics in dietary-induced obesity.

Recently, high-throughput analyses such as genomics, proteomics, and metabolomics have been performed to comprehensively investigate the obesity-related metabolic disorders and better understand the biochemical mechanisms for these conditions. In this study, we selected *L. plantarum* FRT4, a strain isolated from local yogurt without carrying any antimicrobial-resistant genes, which makes it safe as a probiotic candidate. In addition, FRT4 had good acid and bile salt tolerance and lowered cholesterol *in vitro*. The aim of this study was to reveal the multifaceted efficacy of probiotic in preventing obesity in mice fed with HFD *via* combining high-throughput sequencing of gut microbiota and liver metabolomics. By broadening our appreciation of mechanisms involved in the interactions between the microbiota and host, we will be in a better position to provide new avenues for treating dietary-induced obesity.

## Materials and methods

### Preparation of the L. plantarum FRT4 strain

*L. plantarum* FRT4 was isolated from a kind of local yogurt in Xinjiang province, China, identified by 16S rRNA sequencing, and stored in Chinese General Microorganism Collection Center CGMCC under the number of 17955. The strain was propagated in MRS medium at 37°C for 24 h. The strain was propagated in MRS medium at 37°C for 24 h. Cells were harvested by centrifugation and washed three times with sterile saline buffer (0.9%), and then the cells were suspended in saline buffer to the concentration of 1 × 10^9^ and 1 × 10^10^ CFU mL^−^^1^, respectively. Fresh bacterial suspensions were prepared daily for gavage feeding.

### Animal experiments

Thirty-six female Kunming mice aged 7 weeks were purchased from Vital River Laboratory Animal Technology Co. Ltd (Beijing, China) and housed in cages under a 12-h light–dark cycle. After 1 week of adaptation, the mice were randomly divided into two groups: the control group (*n* = 9, CT) fed with a regular chow diet throughout the experiment, and the experiment group (*n* = 27) fed with HFD according to the reported literature with slight modifications (18). Specifically, HFD was composed of 67% (w/w) normal chow, 10% lard, 20% sucrose, 2.5% cholesterol, and 0.5% sodium cholate (Keao Xieli Feed Co., Ltd, Beijing, China). After eight weeks, the HFD-fed mice were randomly divided into three subgroups (*n* = 9 per each group): HF, HF4L, and HF4H groups. The mice in the CT and HF group were orally administered 0.2 mL of sterilized saline (0.9%) once daily, and other two of the subgroups having the HFD diet were treated daily with 0.2 mL of high (HF4H; 1 × 10^10^ CFU/mL) and low (HF4L; 1 × 10^9^ CFU/mL) dosage of *L. plantarum* FRT4, respectively. After 8 weeks of gavage treatment, all of the mice were fasted for 6 h and then sacrificed for serum and tissue collection. The cecal content, the liver, the fat, and colon tissues were precisely dissected, collected, and weighed under aseptic conditions. The blood samples were centrifuged (4°C, 3,000 rpm, 10 min) to obtain the serum. A portion of fresh livers were immediately fixed at room temperature with 4% buffered formalin. All samples were frozen quickly with liquid nitrogen and stored at −80°C for further use. Animal Care and Use Committee of the Institute of Feed Research of Chinese Academy of Agricultural Sciences (CAAS), and protocols were approved by the Laboratory Animal Ethical Committee and its Inspection of the Feed Research Institute of CAAS (AEC-CAAS-20090609).

### Biochemical analyses in the serum and liver

Liver tissues were washed with ice-bold buffered saline, weighed, and homogenized in saline with a homogenizer. The homogenates were then centrifuged (4°C, 3,000 rpm, 20 min) for biochemical analysis. The concentrations of total cholesterol (TC), triglyceride (TG), alanine aminotransferase (ALT), aspartate aminotransferase (AST), high-density lipoprotein cholesterol (HDL-C), low-density lipoprotein cholesterol (LDL-C), and glucose (GLU) in the liver, and serum were performed using commercial kits (Nanjing Jiancheng Bioengineering Institute, Nanjing, China) by an automatic biochemical analyzer 7080 (Hitachi, Ltd., Tokyo, Japan).

### Liver histology

Formalin-fixed liver tissues were dehydrated and embedded in paraffin wax. Slices of liver tissue (thickness of 4–5 μm) were prepared, stained with hematoxylin and eosin (H&E), and then examined under a microscope at 400-fold magnification for further histological analysis (Olympus, Co., Tokyo, Japan).

### Real-time quantitative PCR

Total RNA was extracted from liver and colon tissues, respectively, using Trizol reagent (Invitrogen, Carlsbad, CA, USA). Reverse transcription of cDNA synthesis was performed using an oligo(dT)_18_ primer and Transcript RT kit (Transgen, Beijing, China). The primer sequences for target genes are listed in Table 1. RT-PCR was performed using SYBR Green Master Mix (Thermo Fisher Scientific, Sunnyvale, CA, USA) in a 7500 Fast Real-time PCR system (Applied Biosystems, Carlsbad, CA, USA). The thermal cycling conditions were 95°C for 10 sec, followed by 40 cycles of 95°C for 15 sec (denaturation), and 60°C for 1 min (annealing extension). β-Actin was selected as the internal control and relative gene expression levels were analyzed using the 2^‒ΔΔCt^ method with the CT group (mean) as the reference.

**Table 1:** The sequences of primers used for real-time quantitative PCR

Gene	GenBank no.	Primers sequence (5′→3′)
*ZO-1*	D14340.1	F: GGGCCATCTCAACTCCTGTA
		R: AGAAGGGCTGACGGGTAAAT
*Occludin*	U49185.1	F: ACTATGCGGAAAGAGTTGACAG
		R: GTCATCCACACTCAAGGTCAG
*Claudin-1*	NM_016674.4	F: GAATTCTATGACCCCTTGACCC
		R:TGGTGTTGGGTAAGAGGTTG
*TLR4*	NM_021297.3	F: CTGTTCCTCCAGTCGGTCAG
		R: CGTCGCAGGAGGGAAGTTAG
*IL-6*	NM_031168	F: CCAGTTGCCTTCTTGGGACT
		R: GGTCTGTTGGGAGTGGTATCC
*β-Actin*	NM_007393.5	F: TGTCCACCTTCCAGCAGATGT
		R: AGCTCAGTAACAGTCCGCCTAGA

**Table 2:** Effects of FRT4 on the growth performance and biochemical parameters of serum and liver in mice

Items	CT	HF	HF4L	HF4H
**Growth performance**				
Calorie intake (kcal/each/d)	17.57 ± 0.37	22.73 ± 0.49**	22.69 ± 0.29	22.35 ± 0.57
Initial body weight (g/each)	42.20 ± 0.38	47.88 ± 0.55**	47.75 ± 0.44	47.89 ± 0.19
Final body weight (g/each)	44.12 ± 0.41	53.20 ± 0.81**	50.18 ± 0.63^#^	49.11 ± 0.43^#^
Body weight gain (g/each)	1.92 ± 0.13	5.31 ± 0.45**	2.49 ± 0.35^#^	1.12 ± 0.22^#^
Liver weight (g/each)	1.70 ± 0.17	1.97 ± 0.15**	1.65 ± 0.68^#^	1.84 ± 0.18
Fat weight (g/each)	3.84 ± 1.62	8.03 ± 1.92**	6.81 ± 1.58	5.30 ± 1.89^#^
**Serum biochemistry**				
ALT (U/L)	3.13 ± 0.30	14.63 ± 1.10**	10.63 ± 0.46^##^	9.75 ± 0.65^##^
AST (U/L)	56.13 ± 2.03	53.38 ± 2.31	57.38 ± 2.50	55.38 ± 2.45
GLU (mmol/L)	3.58 ± 0.22	3.79 ± 0.29	2.76 ± 0.21^#^	2.61 ± 0.15^##^
TC (mmol/L)	0.58 ± 0.03	1.64 ± 0.11**	0.89 ± 0.09^##^	1.02 ± 0.09^##^
TG (mmol/L)	0.39 ± 0.04	0.58 ± 0.03**	0.35 ± 0.02^##^	0.33 ± 0.02^##^
HDL-C (mmol/L)	0.30 ± 0.03	0.82 ± 0.04**	0.44 ± 0.03^##^	0.49 ± 0.03^##^
LDL-C (mmol/L)	0.12 ± 0.01	0.14 ± 0.01	0.19 ± 0.01^##^	0.13 ± 0.01^#^
**Liver biochemistry**				
ALT (U/L)	137.12 ± 13.26	716.18 ± 29.78**	430.60 ± 36.76^##^	562.70 ± 30.56^##^
GLU (mmol/L)	11.11 ± 0.60	10.86 ± 0.48	10.86 ± 0.73	8.78 ± 0.69^#^
TG (mmol/L)	1.05 ± 0.05	2.23 ± 0.24**	1.76 ± 0.14^#^	1.43 ± 0.13^##^
HDL-C (mmol/L)	0.04 ± 0.02	0.05 ± 0.01	0.05 ± 0.01	0.06 ± 0.01
LDL-C (mmol/L)	0.09 ± 0.01	0.08 ± 0.01	0.08 ± 0.01	0.10 ± 0.01^#^

Statistical analysis was performed by using one-way analysis of variance with Duncan’s multiple comparison test. Data represent the mean ± SE of each group. *P < 0.05

** *P* < 0.01 compared with the CT group;

# *P* < 0.05

## *P* < 0.01 compared with the HF group.

### Analysis of gut microbiota

Genomic DNA was extracted from cecal samples using the QIAamp DNA Stool Mini Kit (Qiagen, Hilden, Germany). The 16S rRNA gene V3-V4 variable region was PCR-amplified with the universal primer set 341F (5′-CCTAYGGGRBGCASCAG-3′) and 806R (5′-GGACTACNNGGGTATCTAAT-3′). The PCR products were pooled and purified, and sequencing libraries were generated using the Ion Plus Fragment Library Kit (Thermo Scientific, Waltham, MA, USA). The library was then sequenced on an IonS5^TM^ XL platform at Novogene, Beijing, China. All the raw sequencing data were collected into the NCBI Sequence Read Archive database with accession numbers SRR10714265-SRR10714300.

Uparse V7.0.1001 was used to perform operational taxonomic units (OTUs) clustering of sequences with ≥97% similarity (19). Representative sequences for each OTU were screened for further annotation. The Chao1 and abundance-based coverage estimator (ACE) indices were calculated with QIIME V1.9.1 (20) and displayed with R software V2.15.3. Hierarchically clustered heat map and principal component analysis (PCA) based on weighted UniFrac distance matrix were generated by R software V2.15.3. The linear discriminant analysis (LDA) effect size (LEfSe) analysis was employed to identify the significant differences between groups (LDA > 2) by LEfSe software.

### Analysis of liver metabolites

Liver tissues were homogenized, centrifuged, and subjected to metabolomics analysis. Metabolic profiling of liver samples was performed on an Agilent 1290 Infinity LC ultra-high pressure liquid chromatograph (UHPLC) system (Agilent, Santa-Clara, CA, USA) coupled with quadrupole time-of-flight mass spectrometry (UHPLC-Q-TOF/MS). Positive and negative ion mode electrospray ionization (ESI) was used for metabolite detection. After separation by UHPLC, a triple TOF 5600 Mass spectrometer (AB SCIEX, Framingham, MA, USA) was used for MS analysis. The raw UHPLC-Q-TOF/MS data and statistical analysis were referred to the reported literature (21). All differentially abundant metabolites were queried against the online Kyoto Encyclopedia of Genes and Genomes (KEGG) database (http://www.genome.jp/kegg/) to obtain their COs and were then mapped to KEGG pathways. The KEGG pathway enrichment analysis was performed to further explore the influence of differentially expressed metabolites using the Fisher’ exact test, considering the whole metabolites of each pathway as a background data set. Only pathways with *P* < 0.05 were considered to be significantly enriched.

### Statistical analysis

All data were expressed as means ± SE for each group. Statistical significance of difference between groups was analyzed by one-way analysis of variance (ANOVA). When dietary treatments were significantly different (*P <* 0.05), means were compared using Duncan’s multiple comparison procedure of SAS software (SAS Institute, Cary, NC, USA). Spearman correlation coefficients between the concentration of liver metabolites and changes in relative abundance of individual genera were calculated using the R package “stats.” The correlation heatmap was drawn by the R package “corrplot.” *P <* 0.05 was considered to be statistically significant.

## Results

### L. plantarum FRT4 intervention alleviated obesity in HFD-fed mice

**Fig. 1:**
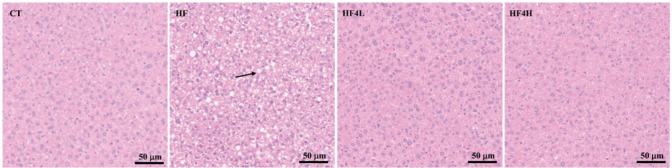
Histological changes of liver sections were measured by H&E staining at 400 × magnification. CT, the control group; HF, the HFD-fed group; HF4L, the HF group fed with a daily FRT4 dose of 5 × 10^8^ CFU/mL; HF4H, the group fed with a daily FRT4 dose of 5 × 10^9^ CFU/mL.

As shown in Table 1, 16 weeks of HFD feeding had induced a significant increase in body weight, body weight gain, liver weight, and fat weight compared with the CT group (*P <* 0.05). FRT4 intervention significantly reduced body weight, body weight gain, liver weight, and fat weight compared with the HF group (*P <* 0.05). Consistently, accumulated lipid droplets and balloon-like structures were observed in liver cells of the HF group, which is the hallmark for hepatic steatosis. After FRT4 treatment, the lipid droplet size was obviously reduced, and the hepatic steatosis gained a remarkable amelioration compared to that of HFD-fed mice (Fig. 1).

### L. plantarum FRT4 intervention affected the biochemical parameters in HFD-fed mice

As shown in Table 1, HFD feeding significantly increased hepatic TG and ALT levels compared with the CT group (*P <* 0.05), which were significantly reduced after FRT4 intervention (*P <* 0.05). No significant differences were found among the four groups of mice for LDL-C and HDL-C in the liver. In addition, serum TG, TC, ALT, and HDL-C levels were significantly higher in the HF group than in the CT group (*P <* 0.05), and FRT4 administration counteracted the HFD effect (*P <* 0.05). FRT4 also significantly lowered the GLU level compared to the HF group (*P <* 0.05). The serum levels of AST and LDL-C were not significantly affected by the HFD feeding or FRT4.

### L. plantarum FRT4 promoted changes in liver metabolites

**Fig. 2:**
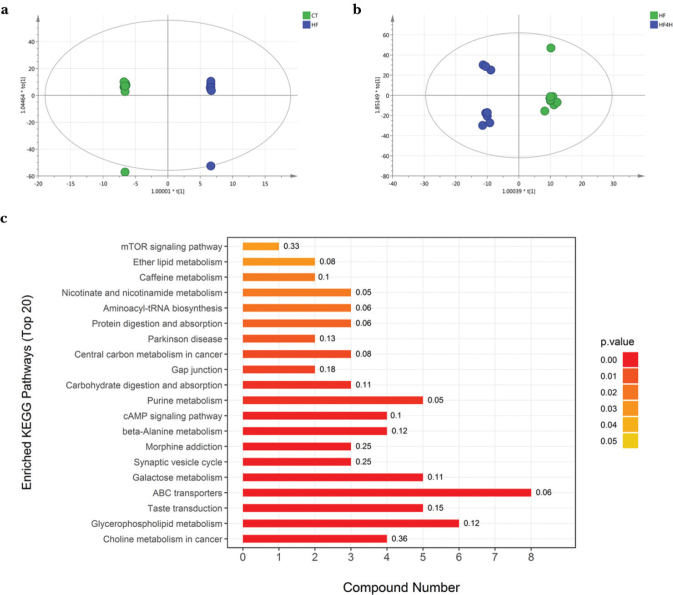
Effect of *L. plantarum* FRT4 on liver metabolites was investigated by nontargeted metabolomics. OPLS-DA score plot analysis under the positive ion mode comparison between HF and CT (a), HF and HF4H (b). Enrichment analysis results of the KEGG pathway between HF4H and HF groups (c). Rich factor is defined as the ratio of the number of differential metabolites to total metabolites in each pathway.

To further explore the lipid-lowering mechanism of FRT4, untargeted metabolomics analysis was performed for liver samples of mice. The orthogonal partial least squares discriminant analyses (OPLS-DA) score plot revealed that HFD feeding induced different clustering changes compared to the CT group (Fig. 2a). Compared to the HF group, FRT4 treatment resulted in distinct clustering and complex systemic changes in the livers (Fig. 2b). According to the KEGG reference pathways associated with the lipid metabolism, indicators with significantly discriminative power were the “Glycerophospholipid metabolism,” “Choline metabolism in cancer,” and “Galactose metabolism,” which showed significant enrichment with FRT4 treatment (Fig. 2c).

We found abnormal aspects of lipid metabolism in HFD-fed mice, such as glycerophospholipid metabolism, which provides novel insight into the role of lipids in obesity. As shown in Table 3, six intermediates, including choline, cytidine 5′-diphosphocholine (CDP-choline), glycerophosphocholine, phosphorylcholine, sn-glycerol 3-phosphoethanolamine, and O-phosphoethanolamine, were significantly greater in HFD-fed mice (*P <* 0.05), while phosphatidylcholine (PC) (16:0/16:0) was significantly decreased (*P <* 0.05). Compared to the HF group, FRT4 intervention significantly decreased the amounts of choline, glycerophosphocholine, and phosphorylcholine (*P <* 0.05). However, there was no significant difference in PC (16:0/16:0) between the HF and FRT4-treated groups. In addition, HFD induced decreased the trimethylammonium cation (TMA cation) compared to the CT group, which was further decreased after FRT4 intervention. Moreover, on these results, we observed that several metabolites were also significantly changed, including the levels of pyrimidines, purines, nicotinamide, and amino acids (data not shown).

**Table 3:** Metabolites’ changes among different groups

Metabolites	RT (s)	m/z	Fold change		
			HF vs. CT	HF4L vs. HF	HF4H vs. HF
Choline	780.035	104.1058352	1.38*	0.83*	0.82*
Glycerophosphocholine	862.561	258.1094388	2.13*	–	0.45*
CDP-choline	878.204	489.1135327	2.13*	–	0.45+
Phosphorylcholine	717.05	242.0794821	2.39*	0.77+	0.74*
sn-glycerol 3-phosphoethanolamine	788.63	198.0517674	1.94*	–	0.63*
*O*-phosphoethanolamine	915.7575	142.0254256	1.71*	–	-
PC (16:0/16:0)	315.6605	756.5520133	0.46*	–	-
TMA cation	760.846	146.1166382	0.63*	0.57+	0.43*

Fold change is expressed as the ratio between two groups. Metabolites with both VIP > 1 and *P <* 0.05 are considered to be significantly different and denoted with asterisks, and those with VIP > 1 and 0.05 < *P <* 0.1 are considered as differential and denoted with plus signs.

### L. plantarum FRT4 changed the expression of key genes involved in intestinal barrier function and low-grade chronic inflammation

**Fig. 3:**
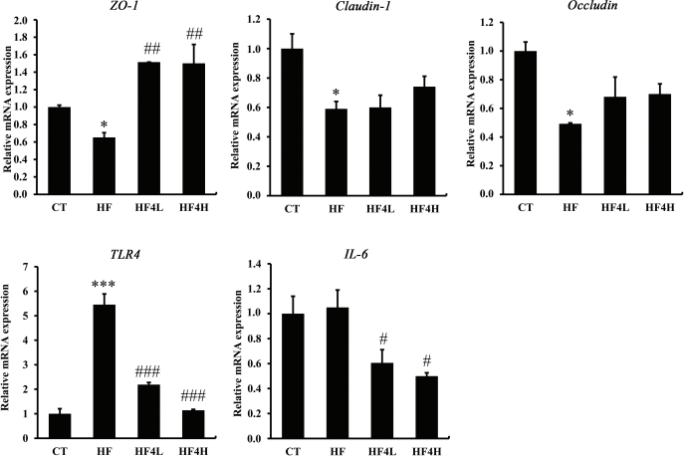
Effects of FRT4 on gut barrier dysfunction and low-grade chronic inflammation in the HFD-fed mice. The data are shown as the means ± SE (*n* = 4 in each group). Statistical analysis was conducted by using one-way analysis of variance with Duncan’s multiple comparison test. Significant differences between HF and CT are indicated as * for *P* < 0.05, ** for *P* < 0.01, and *** for *P* < 0.001. Significant differences of HF versus HF4L and HF4H are indicated as ^#^ for *P* < 0.05, ^##^ for *P* < 0.01, and ^###^ for *P* < 0.001.

Considering that HFD may affect epithelial integrity, we studied the influence of FRT4 on the expression of three tight junction-related genes in the colon. Intestinal barrier dysfunction, such as the disruption of tight junction proteins zonula occludens-1 (ZO-1), occludin and claudin-1 that lead to impaired gut permeability and low-grade systemic inflammation (5), can lead to the development and progression of NAFLD. As shown in Fig. 3, in comparison with the HFD group, the mRNA expression levels of ZO-1, *occludin*, and *claudin-1* were markedly increased but had no significant difference. Toll-like receptor 4 (*TLR4*) and *IL-6* are an important endotoxin receptor and a proinflammatory factor, which were lowered after FRT4 administration. These results indicated that FRT4 intervention improved gut barrier integrity and attenuated inflammation.

### L. plantarum FRT4 intervention regulated gut microbiota composition in HFD-induced gut dysbiosis

**Fig. 4:**
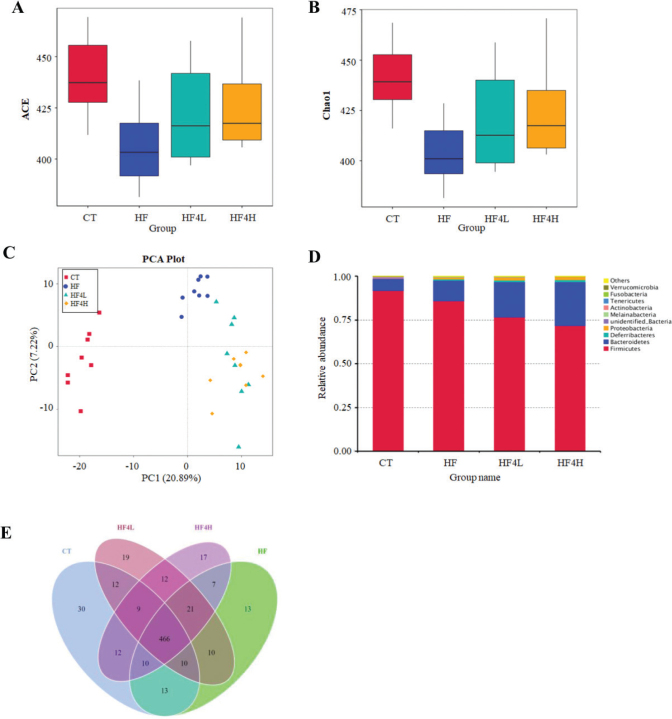
Effects of *L. plantarum* FRT4 treatment on gut microbiota composition. (a) ACE. (b) Chao1. (c) PCA plot. (d) Microbial composition at the phylum level (top 10). (e) Venn diagram representation of the number of OTUs at the genus level from the CT, HF, HF4L, and HF4H groups. *n* = 8 in each group.

**Fig. 5:**
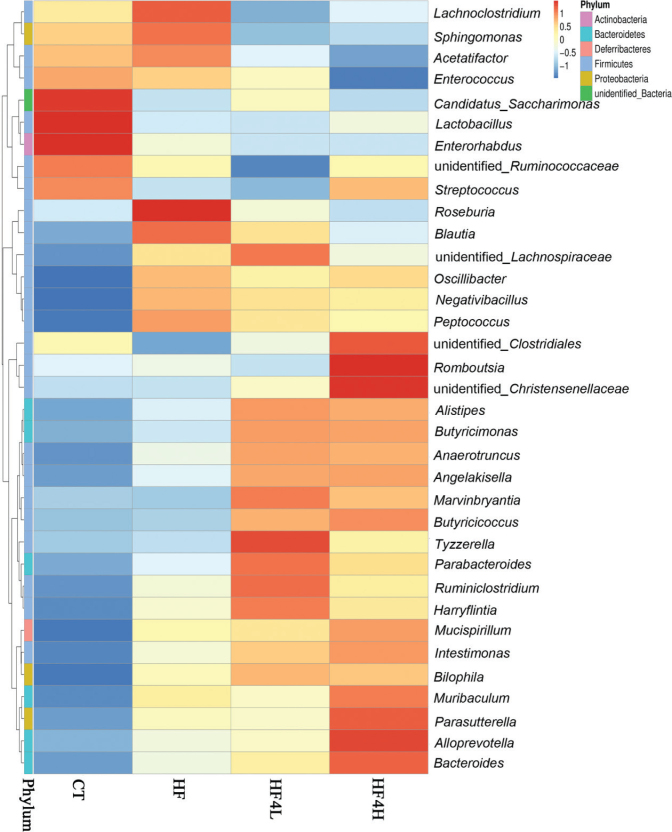
Heatmap showing the hierarchical clustering of the bacterial genus profiles of the 35 most abundant OTUs in the CT, HF, HF4L, and HF4H groups. *n* = 8 in each group.

The α-diversity of the gut microbiota, including ACE (Fig. 4a) and Chao 1 (Fig. 4b) rarefaction indices, showed that FRT4 administration increased the microbial richness and diversity. PCA score plot reflecting the β-diversity showed that the gut microbiota structure in the HF group presented a structural shift from the CT group, and FRT4 administration presented a structural shift from the HF group (Fig. 4c). Compared to the HFD group, FRT4-treated groups significantly increased the relative abundance of *Bacteroidetes* and lowered number of *Firmicutes* (Fig. 4d). At the genus level, the Venn diagrams showed that the 466 OTUs were identical among the four groups, showing a strong core microbiota (Fig. 4e). The CT group demonstrated the most unique genus (30 OTUs) followed by the HF4L group (19 OTUs), HF4H group (17 OTUs), and HF group (13 OTUs). We further identified 35 genus taxa that were altered by FRT4 intervention (Fig. 5a). Compared to the HF group, FRT4 intervention selectively enriched the *Bacteroides*, *Alistipes*, *Intestinimonas*, *Butyricimonas*, *Butyricicoccus,* and *Lactobacillus* and suppressed *Roseburia* and *Blautia*. In addition, *Akkermansia* and *Odoribacter* reappeared after FRT4 treatment in HFD-fed mice (data not shown).

Distinct community profiles were then compared at all taxonomic-based levels among samples. LEfSe (LDA > 2) was performed to analyze the relative abundance of significantly different phylotypes (Fig. 5b). Based on the comparison among the CT, HF, and HF4H group, 103 phylotypes were identified as biomarkers for distinguishing gut microbiota under different treatments. The HF4H group demonstrated the highest phylotypes (57 phylotypes) followed by the CT group (32 phylotypes) and HF group (14 phylotypes). The abundance of 23 genera differed significantly between FRT4-treated mice and the HFD-fed mice. The amount of phylotypes *Lachnoclostridium*, *Roseburia*, and *Oscillibacter* was higher in the HF group, while the phylotypes *Bacteroides*, *Parabateroides*, *Anaerotruncus*, *Alistipes*, *Intestinimonas*, *Butyicicoccus*, and *Butyricimonas* were higher in the HF4H group. These findings suggested that FRT4 administration ameliorated gut dysbiosis by manipulating the microbial composition in the HFD-fed mice.

### Correlation between gut microbiota and the liver metabolites

**Fig. 6:**
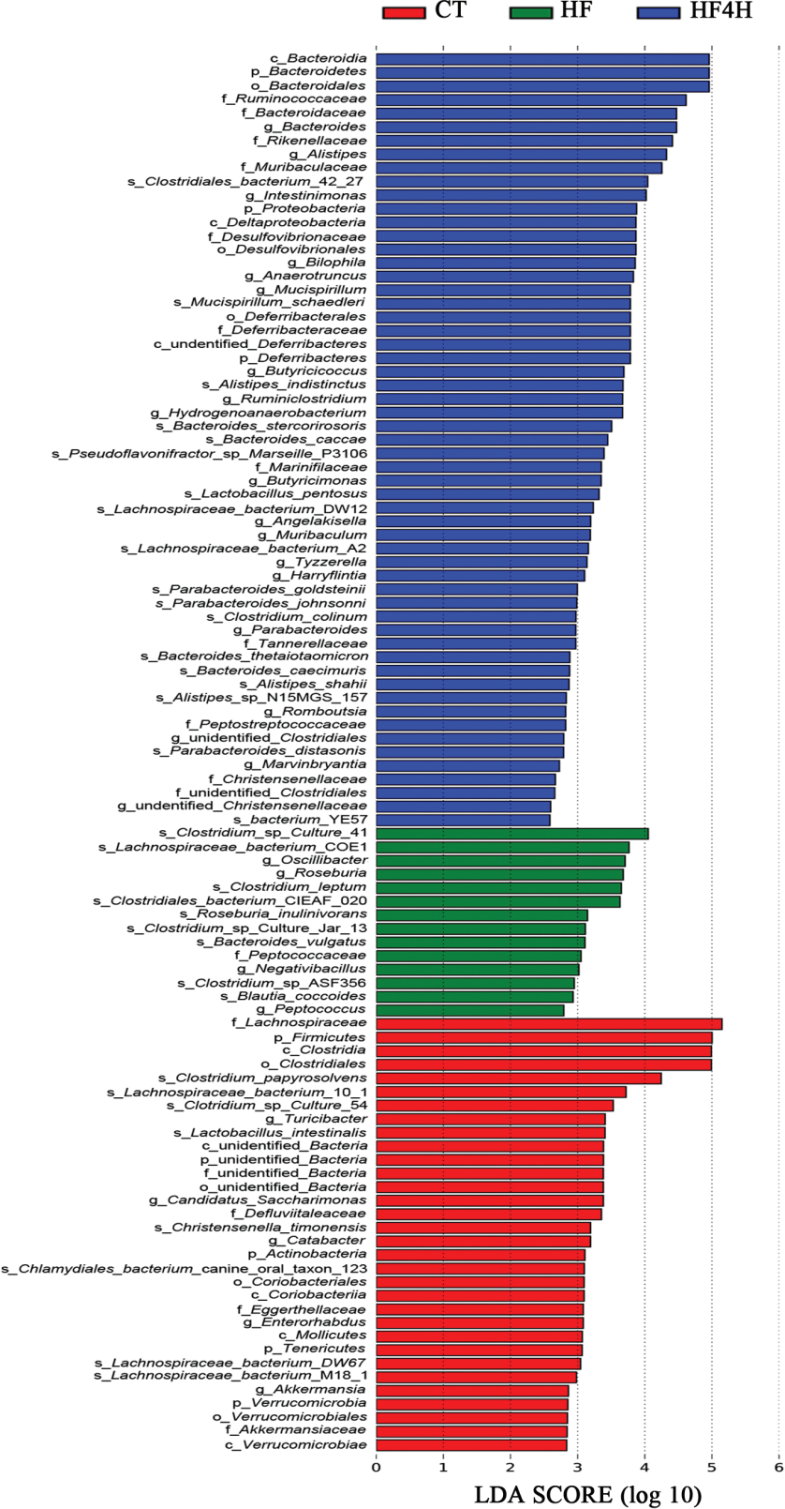
LEfSe identified the gut microbiota phylotypes with a statistically significant difference in abundance among the CT, HF, and HF4H groups with LDA > 2. *n* = 8 in each group.

The correlations between the key microbial phylotypes responding to FRT4 treatment and liver metabolites-related parameters were studied (Fig. 6). It was found that the changes of *Oscillibacter* and *Roseburia* were positively associated with those of choline and glycerophosphocholine (*P <* 0.05). The change in *Akkermansia* was negatively associated with choline and glycerophosphocholine. *Butyricicocus* showed a negative correlation with phosphorylcholine. Interestingly, *Alistipes*, *Intestinimonas*, *Bacteroides, Butyricicoccus, Butyricimonas, and Parabacteroides* showed a negative correlation and *Catabacter* showed a positive correlation with that of the TMA cation (*P* < 0.05).

**Fig. 7:**
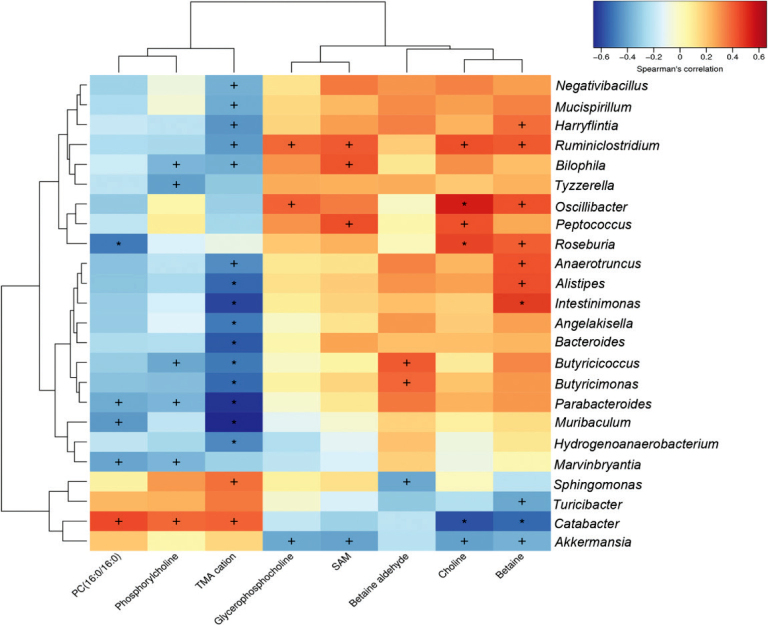
Spearman correlation analysis between the concentration of liver metabolites and changes in relative abundance of individual genus. Significant differences at *P <* 0.05 and *P <* 0.01 are denoted with + and *, respectively.

## Discussion

In this study, we demonstrated that *L. plantarum* FRT4 treatment resulted in reduced body weight gain, liver weight, and fat weight in HFD-induced obesity in mice. In addition to significantly lowering TG and ALT levels in the serum and liver, FRT4 intervention also lowered TC in the serum, which is consistent with the hypocholesterolemic effects of *L. plantarum* WCFS1 (10). Elevated ALT and AST levels in plasma are indicative of liver damage, and particularly, ALT has been widely used as an alternative marker for NAFLD (22). The data suggested that FRT4 improved liver function in mice with HFD-induced obesity.

Choline is an essential nutrient to maintain normal liver function, as well as phospholipids (23), which is predominantly absorbed from the small intestine and completely metabolized in the liver (24). Choline is also an important precursor to PC, the main phospholipid coating very low density lipoprotein (VLDL) particles (24). In our study, elevated choline was observed in the HF group compared to the CT group. The results indicated that HFD induced a reduction in choline metabolism, which could reduce efflux of VLDL from hepatocytes (5). Our research is consistent with the studies that reducing the bioavailability of choline could result in NAFLD both in mice (25) and in humans (26). Leung et al. reported that more choline is associated with greater risks of having hepatic steatosis, nonalcoholic steatohepatitis (NASH), and lobular inflammation (5). A previous study also pointed out that plasma-free choline levels are positively related to the grade of liver steatosis and fibrosis (27). Moreover, increased choline in the liver of the HF group may be caused by increased intestinal permeability and further damage to the epithelial barrier from gut microbiota dysbiosis (5, 28). In addition, HFD leads to the reduction of choline metabolism, which can reduce efflux of VLDL from hepatocytes and promote inflammation (5). However, the result was not in accordance with a previous study that there was a reverse significant association between choline intake and the risk of NAFLD (29). FRT4 intervention improved the intestinal barrier effect through upregulation of the gene expression of ZO-1. The dysfunction of ZO-1 can lead to impaired gut permeability and low-grade systemic inflammation (5) and further result in NAFLD occurrence and progression. Obesity is characterized by low-grade chronic inflammation, possibly triggered by TLR2 and TLR4 activation (30). TLRs, particularly TLR4, are activated by fatty acids and endotoxinema, leading to activation of nuclear factor-κB and increased release of inflammatory factors (30). Accordingly, the gene expression of *TLR4* was elevated in the HF group and FRT4 intervention decreased the gene expression of *TLR4* and *IL-6*, which is in line with the reports that IL-6 was diminished by the treatment with *L. reuteri* V3401 in adults with metabolic syndrome and *L. plantarum* HFY09 alleviated inflammation by decreasing the levels of pro-inflammatory factors IL-6, IL-1β, and TNF-α in ethanol-induced liver injury in mice (31, 32). These results indicated that FRT4 intervention enhanced choline metabolism, suppressing inflammatory responses by decreasing the expression of pro-inflammatory biomediators.

Our research consistently found that choline-related metabolites such as glycerophosphocholine and phosphorylcholine were higher in the model group than those in the control. Excess choline, glycerophosphocholine, phosphorylcholine, and CDP-choline were also positively correlated with fat accumulation, which was consistent with a previous study that an elevated level of glycerophospholipid metabolites in obese mice was positively correlated with fat accumulation (33). FRT4 treatment significantly reduced glycerophosphocholine induced by HFD, which is not in accordance with a previous report that glycerophosphocholine was increased after the treatment of *L. plantarum* NCIMB8826 (34). Our results may be explained by the finding that glycerophosphocholine was metabolized to choline, with this choline serving as the substrate for renewed synthesis of PC (35). Although there is no significant difference in PC between the FRT4 with HFD-fed and HFD-fed mice, PC still tended to be higher in FRT4 with the HFD-fed group than in the HFD-fed group alone. Interestingly, we observed the decrease of TMA cations in the HF group, which was further decreased after FRT4 intervention. Most of the TMA produced by the gut microbiota metabolizing choline and L-carnitine is absorbed into the blood and rapidly oxidized to TMAO by the hepatic enzyme (36). Trimethylamine (TMA) and trimethylamine *N*-oxide (TMAO) can promote the development of fatty liver disease (37). TMAO and choline with high concentrations were correlated with an advanced cardiometabolic risk profile (38). FRT4 intervention decreased the level of TMA cations and choline in the liver (Table 3). Restoration of HFD-induced changes in choline, glycerophosphocholine, and phosphorylcholine by FRT4 may contribute to improve the lipid profile given the roles of glycerophospholipid components in maintaining lipid homeostasis.

FRT4 intervention increased the relative abundance of *Bacteroides*, *Anaerotruncus*, *Parabacteroides*, *Alistipes*, *Intestinimonas*, *Butyicicoccus*, and *Butyricimonas* in the HFD-treated mice. *Bacteroides* were negatively correlated with energy intake and adiposity (39). *Anaerotruncus*, *Parabacteroides*, *Alistipes*, *Intestinimonas*, and *Butyricimonas* were reported to be negatively associated with obesity (40). *Alistipes* at a relatively high proportion could effectively suppress inflammation by inhibiting LPS-induced TNF-a release (34). Short-chain fatty acid (SCFA) acetate, propionate, and butyrate were derived from the microbial fermentation of fibers, linking host nutrition to maintenance of intestinal homoeostasis. As important fuels for intestinal epithelial cells (IEC), microbial SCFA regulates the functions of IEC to affect gut motility, enhance the gut barrier functions and host metabolism, and reduce intestinal inflammation (41, 42). Increasing SCFA production could be a valuable strategy for preventing type 2 diabetes, obesity, and gastrointestinal dysfunction (42). Due to the production of SCFA, *Intestinimonas* had the ability to provide energy to intestinal cells and protecte the intestinal barrier (43). In the gut lumen, *Butyricicoccus* and *Butyricimonas* were producers of butyrate that may play critical roles in metabolic improvements (44) and were reported to reduce dietary energy (45). In addition, FRT4 treatment reduced the abundance of *Oscillibacter*. Several studies showed that *Oscillibacter* might be involved in HFD-induced intestinal dysfunction (46) and remained associated with the gut permeability (47). Interestingly, *Blautia* and *Roseburia*, reported as butyrate producers, were enriched in the HF group but were less abundant with FRT4 treatment. *Blautia* was reported to have significantly positive correlations with triglyceride and cholesterol (48). Recent studies found that in diabetic children or obese mice, there were low amount of *Roseburia* and high abundance of *Blautia* (49, 50). The functions of *Roseburia* and *Blautia* need further confirmation. These results showed that FRT4 intervention reshaped the gut microbiota composition and diversity in HFD-fed mice.

The endotoxins and other intestinal bacteria-derived products that reach the liver through the gut-liver axis interface can trigger an adverse innate immune system and inflammatory activity, which further promotes the progression of obesity. The Spearman correlation analysis revealed that *Oscillibacter* and *Roseburia* were positively correlated with choline and glycerophosphocholine. *Oscillibacter* could increase the intestinal permeability, which may account for the excess choline found in the liver (51). SCFA-producing bacteria *Butyricicoccus* showed a negative correlation with phosphorylcholine. SCFA appeared to exert overall beneficial metabolic and antisteatotic hepatic effects (37). *Alistipes*, *Intestinimonas*, *Bacteroides, Butyricicoccus, Butyricimonas, and Parabacteroides* showed a negative correlation with hepatic TMA cations, which indicated that these genera played an important role in regulating TMA conversion from choline in the gut and should be further studied. The close relationships between gut microbiota and liver metabolites further supported our conjecture that FRT4 administration had protective effects against obesity by modulating gut microbiota.

## Conclusions

In the current work, we discovered an interesting phenomenon: a HFD significantly increased choline, glycerophosphocholine, phosphorylcholine, and CDP-choline, indicating dysregulation of glycerophospholipid metabolism. FRT4 intervention alleviated the condition by manipulating gut microbiota as a preliminary point and further affected lipid metabolism in the liver. In addition, these data have identified glycerophosphocholine metabolites as potential novel biomarkers of HFD-induced obesity, increased the predictability of the obesity risk, and confirmed the association of known metabolites with altered gut microbes. Through increased understanding of mechanisms involved in the interactions between the microbiota and host, we will be in a better position to treat dietary-induced obesity.
